# Seeing is believing: methods to monitor vertebrate autophagy *in vivo*

**DOI:** 10.1098/rsob.180106

**Published:** 2018-10-24

**Authors:** Ana Lopez, Angeleen Fleming, David C. Rubinsztein

**Affiliations:** 1 Department of Medical Genetics, University of Cambridge, Cambridge Institute for Medical Research, Wellcome Trust/MRC Building, Cambridge Biomedical Campus, Hills Road, Cambridge CB2 0XY, UK; 2 UK Dementia Research Institute, University of Cambridge, Cambridge Institute for Medical Research, Wellcome Trust/MRC Building, Cambridge Biomedical Campus, Hills Road, Cambridge CB2 0XY, UK; 3 Department of Physiology, Development and Neuroscience, University of Cambridge, Downing Street, Cambridge CB2 3DY, UK

**Keywords:** autophagy, zebrafish, *in vivo*, neurodegeneration, fluorescent probes, *in vivo* assays

## Abstract

Autophagy is an intracellular clearance pathway that delivers cytoplasmic contents to the lysosome for degradation. It plays a critical role in maintaining protein homeostasis and providing nutrients under conditions where the cell is starved. It also helps to remove damaged organelles and misfolded or aggregated proteins. Thus, it is not surprising that defects in this pathway are associated with a variety of pathological conditions, such as neurodegeneration, cancer and infection. Pharmacological upregulation of autophagy is considered a promising therapeutic strategy for the treatment of neurodegenerative and infectious diseases. Studies in knockout mice have demonstrated that autophagy is essential for nervous system function, and data from invertebrate and vertebrate models suggest that the efficiency of autophagic processes generally declines with age. However, much of our understanding of the intracellular regulation of autophagy comes from *in vitro* studies, and there is a paucity of knowledge about how this process is regulated within different tissues and during the processes of ageing and disease. Here, we review the available tools to probe these questions *in vivo* within vertebrate model systems. We discuss how these tools have been used to date and consider future avenues of research.

## 1. Autophagy cell biology

**Figure 1.**
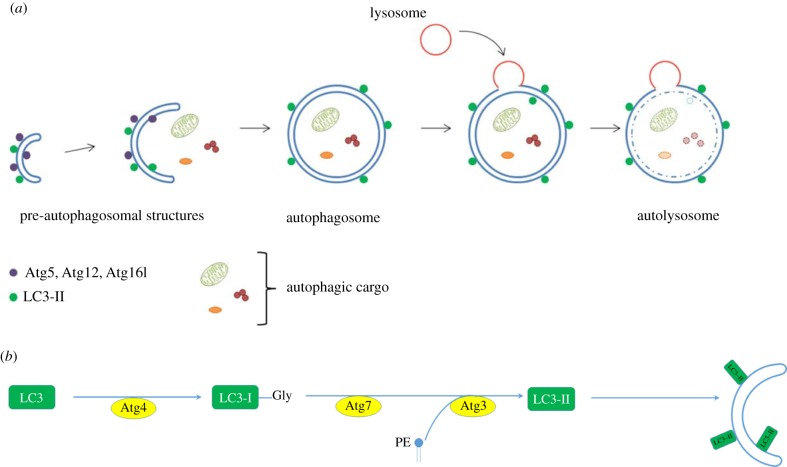
(*a*) Autophagosome formation. Schematic of autophagosome formation and degradation: Within the cytoplasm, double-membraned, sac-like structures called phagophores are the first morphologically recognizable autophagic precursors and can be distinguished within cells by the proteins that associate with their membranes. A complex comprising ATG12–ATG5–ATG16L1 proteins enables the conjugation of LC3-II to the membranes. The edges of the phagophore elongate and eventually fuse while engulfing a portion of the cytoplasm. As the phagophore enlarges and approaches closure, the ATG5–ATG12–ATG16L1 complex dissociates from the outer membrane, whereas LC3-II remains associated. The resulting structure is a spherical double-membrane organelle, called the autophagosome. Following closure, autophagosomes are trafficked by dynein motors along microtubules to the perinuclear region where they fuse with the lysosomes and their contents are degraded. (*b*) Lipidation of LC3-II. During autophagosome formation, LC3 (and other ATG8 ubiquitin-like family proteins) are conjugated to the lipid PE in autophagosome membranes. This lipidation requires a protease and two ubiquitin-like conjugation systems (explained in detail in [1,2]). ATG4 is a cysteine protease which cleaves the C-terminus of LC3 exposing a glycine residue. This first cleaved form of LC3 is called LC3-I. A further reaction then occurs involving a complex of ATG12–5 and ATG16L1, which together act as an E3-like ligase. This determines the site of LC3 lipidation and assists the transfer of LC3-I to PE in membranes to form LC3-II. ATG8/LC3 proteins may assist in the expansion and closure of autophagosomal membranes, in autophagosome-lysosome fusion and inner autophagosomal membrane degradation.

In the initial steps of autophagy, a double-membraned, cup-shaped precursor (called the phagophore) forms within the cytoplasm. The phagophore expands, engulfing substrates as it does so, and eventually the edges fuse to form a double-membraned vesicle, the autophagosome. This traffics along microtubules to the lysosome, with which it fuses resulting in the degradation of the autophagic contents (figure 1). Autophagy is controlled through a conserved family of approximately 30 core genes that encode the autophagic machinery, termed the AuTophaGy-related (*atg*) gene family [3]. The *atg* genes were originally discovered in yeast; mutations in these genes resulted in an inability to survive nutrient deprivation conditions. Most of these genes have vertebrate homologues that are named after their yeast counterparts. Interestingly, many of the yeast genes have more than one vertebrate homologue [3,4], which may contribute to either redundancy or to additional functional diversity.

To follow this process *in vivo*, it is necessary to label and visualize the phagophores and autophagosomes. However, few proteins are uniquely associated with autophagic vesicles and their precursors, with only one protein family (including LC3-II) known to label autophagic structures both prior to and after fusion with the lysosome. LC3 is one of several vertebrate homologues of ATG8. Mammalian cells have six ATG8 orthologues; the MAP1-LC3 (LC3) and GABARAP subfamilies (microtubule-associated protein 1 light chain 3 and GABA(A) receptor-associated protein families respectively), while zebrafish have eight (table 1). During autophagosome formation, these ATG8-family proteins are conjugated to the lipid phosphatidylethanolamine (PE) in autophagosomal membranes. This lipidation requires a protease and two ubiquitin-like conjugation systems [1,2] (figure 1). ATG4 is a cysteine protease that cleaves the C-terminus of LC3, exposing a glycine residue. This first cleaved form of LC3 is called LC3-I. A further reaction then occurs involving a complex of ATG proteins that act as an E3-like ligase. This determines the site of LC3 lipidation and assists the transfer of LC3-I to PE to form LC3-II [1].

**Table 1.** Comparison of zebrafish and human orthologues of ATG8.

zebrafish gene	description	Ensembl ID	human orthologue	percentage identity to human orthologue
Map1lc3a	microtubule-associated protein 1 light chain 3 alpha	ENSDARG00000033609	MAP1LC3A	85.95%
Map1lc3b	microtubule-associated protein 1 light chain 3 beta	ENSDARG00000101127	MAP1LC3B2	92.62%
Map1lc3c	microtubule-associated protein 1 light chain 3 gamma	ENSDARG00000100528	MAP1LC3C	65.47%
Map1lc3cl	microtubule-associated protein 1 light chain 3 gamma, like	ENSDARG00000075727	no human orthologue	(58.73% identity to zebrafish map1lc3c)
gabarapa	GABA(A) receptor-associated protein a	ENSDARG00000035557	GABARAP	93.44%
gabarapb	GABA(A) receptor-associated protein b	ENSDARG00000052082	GABARAP	75.66%
zgc:92606	not annotated	ENSDARG00000040971	GABARAPL1	58.97%
gabarapl2	GABA(A) receptor-associated protein like 2	ENSDARG00000027200	GABARAPL2	96.58%

**Figure 2.**
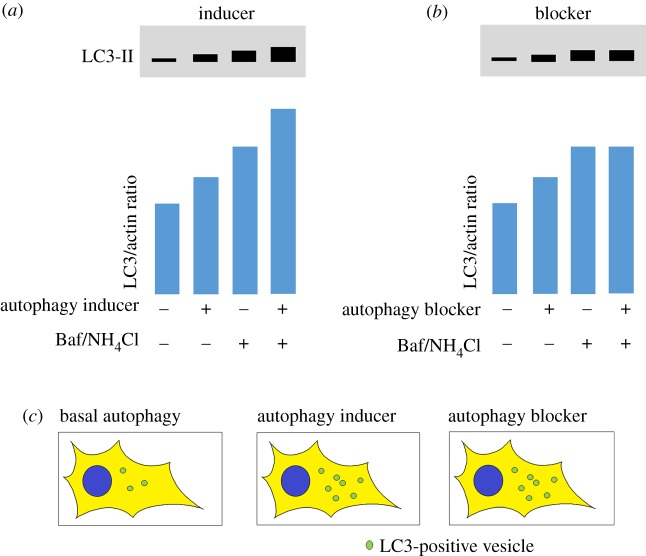
Schematic diagram of conventional methods to measure rates of autophagy. (*a*,*b*) Western blots for LC3-II: Measuring LC3 lipidation by western blotting is one of the best-established methods for measuring autophagic flux. However, care must be taken in interpreting increases in LC3 levels as this may occur as a result of an increase in autophagosome formation (upregulation) or a blockage in clearance. To discriminate between these two scenarios, assays should be performed in basal conditions and in the presence of an agent that prevents lysosomal degradation such as bafilomycin A1 (Baf) or ammonium chloride (NH_4_Cl). (*a*) When autophagy is induced, LC3-II levels increase as more autophagosomes are formed. In the presence of a lysosomal blocker, LC3-II levels increase further because increased autophagosome formation still occurs, but autophagosomes cannot be cleared and therefore build up within the cell. (*b*) In some conditions when autophagy is blocked (for example, if fusion with the lysosome is prevented), LC3-II levels can also increase because autophagosomes may form but are not degraded. In this scenario, when LC3-II levels are measured in the presence of Baf or NH_4_Cl, LC3-II levels are unchanged. The difference in patterns between (*a*) and (*b*) can be used to discriminate between autophagy induction and blockage. (*c*) When LC3-labelled vesicles (puncta) are measured within cells with a single fluorophore (e.g. cells expressing GFP-tagged LC3 or immunofluorescence labelling of the endogenous protein), an increase in puncta can be observed both in autophagy inducing and autophagy blockage conditions. N.B. Commercially available antibodies with cross-reactivity to zebrafish LC3 are widely available from suppliers such as from Novus Biologicals (used in [7–9]) and Cell Signaling Technology (used in [10]).

Since lipidated ATG8 proteins (such as LC3-II) are the only proteins which associate with pre-autophagosomal structures, autophagosomes and autolysosomes, they are widely accepted as being the best marker to distinguish autophagic vesicles from other cellular membranes [5,6]. Measuring LC3 lipidation by western blotting, counting the number of LC3 vesicles by immunofluorescence or with fluorescently tagged LC3 expression constructs, and detecting the degradation of long-lived proteins or damaged organelles are the most commonly used methods for monitoring autophagy [5,6]. However, care must be taken in interpreting increases in LC3 levels as this may occur as a result of an increase in autophagosome formation (upregulation) or a blockage in clearance. In the latter scenario, autophagosomes are not degraded typically due to failure to fuse with lysosomes or due to an increase in lysosomal pH, which thereby inactivates the degradative enzymes (figure 2).

The majority of studies using these biochemical or fluorescent detection methods have only provided a snapshot of autophagic activity within a single tissue at a single time. Many studies have reported that basal levels of autophagy differ between different tissues, and we do not fully understand how these different rates are affected by pharmacological upregulation or disease pathology. Since upregulation of autophagy is considered to be a promising therapeutic strategy for the treatment of a range of disorders, including neurodegeneration, infectious disease and cancer [11,12], it is vital that we understand how potential therapies act in different tissues, and this can only be done by *in vivo* analysis. Similarly, to understand the role of autophagy in the pathogenesis of disease, it is important to study this process in the whole animal to investigate tissue-specific changes in flux, the difference in flux between young and old animals, and cell-autonomous versus non-cell-autonomous effects. In recent years, various transgenic reporters have been developed which may be useful to improve our understanding of autophagy *in vivo*. Together with advances in imaging such as CLEM (correlated light and electron microscopy) and lightsheet microscopy, we now have the tools to interrogate this process in living vertebrate animals. Although such imaging is in its infancy, here we review the available tools and highlight the future possibilities for studying autophagy *in vivo*.

## 2. Single fluorophore probes

The use of a fusion construct comprising green fluorescent protein (GFP) tagged to LC3 was the first approach to examine autophagy *in vivo* in vertebrates and provided novel insights about its regulation in both physiological and pathological conditions. The overexpression of Atg8 homologues fused with GFP had been previously described in other species, such as yeast, *Caenorhabditis elegans, Dictyostelium discoideum, Drosophila melanogaster* and *Arabidopsis thaliana* [13–16].

GFP-LC3, like endogenous LC3, becomes conjugated to the phagophore and remains on the membrane after the complete closure of the autophagosome. Autophagosomes labelled with GFP-LC3 are evident as puncta or ring-like structures by fluorescence microscopy [17–19]. GFP-LC3 can also be found on the membrane of autolysosomes but to a lesser extent. The fluorescent signal of these autolysosomes is weaker and therefore distinguishable from bright autophagosomes [17].

The generation of transgenic mice expressing GFP-LC3 under the control of a ubiquitous promoter has allowed the post-mortem examination of GFP-LC3 localization by high-resolution microscopy and in almost all tissues [20]. The overexpression of GFP-LC3 in mice permits not only qualitative but quantitative analysis of autophagosome numbers and does not affect endogenous autophagy, since the endogenous ratio of LC3II/LC3-I is maintained. Post-mortem analysis of tissues from this transgenic mouse have been used to measure autophagosome numbers during development [21], under starvation conditions [20], or in different disease states such as amyotrophic lateral sclerosis (ALS) [22], polycystic kidney disease [23] and cerebral ischaemia [24]. In addition, primary cultures from these mice have been used for *ex vivo* real-time observations of GFP-LC3 positive autophagic structures [20,25].

An important consideration in the analysis of such reporter lines is to determine whether the fluorescent protein is a faithful reporter of the endogenous protein. Kuma and colleagues [26] demonstrated by western blot analysis that the levels of endogenous LC3 and GFP-LC3 protein are organ-dependent rather than uniform. In the brain, the level of expression of GFP-LC3 was comparable with endogenous LC3, whereas in other tissues GFP-LC3 was overexpressed. Importantly, the integration of the GFP-LC3 transgene, upstream of an open reading frame in a pseudogene in the distal region of chromosome 2, did not cause any phenotypic or genetic abnormalities in homozygous mice [26].

Zebrafish are potentially a more tractable model to study autophagy *in vivo* since they are amenable to most forms of fluorescent imaging due to their size and transparency. Furthermore, analysis is not restricted to embryonic stages, as their rapid development permits the analysis of functioning organs in larvae at free-swimming stages. Zebrafish have eight homologues of Atg8 (table 1) with high sequence similarity to their mammalian orthologues. He *et al.* generated the first transgenic zebrafish autophagy reporter lines for expressing GFP-LC3 and GFP-Gabarap under the control of the constitutive cytomegalovirus (CMV) promoter [7]. Both transgenes showed similar expression patterns; expression being especially high in spinal cord, muscle and lens. Similar to mammalian LC3, zebrafish LC3-I conjugates to PE to generate LC3-II. Initial studies reported that LC3-II was only observed in embryos from 24 h post-fertilization (h.p.f.) onwards by western blotting [7]. However, Lee *et al.* detected autophagy at approximately 15 h.p.f., evidenced by the presence of autophagosomes visualized as GFP-LC3 puncta in the CMV:GFP-LC3 transgenic reporter line [27]. The benefit of this model is not only the ability to perform live imaging, but also to examine multiple tissues within the same animal. Imaging of GFP-LC3 transgenic embryos by confocal fluorescence microscopy showed that the GFP-LC3 protein forms few puncta in basal conditions but the number of puncta increase after autophagy upregulation by addition of rapamycin or calpain inhibitors to the embryo medium [7]. The fusion of autophagosomes to the lysosomes can also be detected *in vivo* by adding LysoTracker to the embryo medium [7]. A dramatic increase in the co-localization of LysoTracker red-labelled lysosomes with GFP-LC3 puncta was observed upon the treatment with lysosomal protease inhibitors like pepstatin A or E64d, suggesting that basal autophagic flux is high in these embryonic and early larval stages (2 and 3 d.p.f) [28].

Several studies have exploited the ability to perform *in vivo* imaging in this GFP-LC3 zebrafish line, for example, to study the role of autophagy in blastema formation and regeneration following fin amputation [29], or in the liver to examine autophagic responses to pharmacological manipulation [30]. The ability to perform transient gene knockdown using morpholino oligonucleotides [31] in zebrafish has enabled the rapid analysis of candidate genes in the regulation of different stages of the autophagy pathway. For example, transient silencing of *Hs1bp3*, a phosphoinositide-binding PX domain-containing protein, increased the number of GFP-LC3 puncta visualized directly along the trunk of morphants compared with control embryos, and this increase was greater after chloroquine treatment, suggesting increased autophagic flux *in vivo* [32]. A similar approach was taken to study *spns1*, a putative lysosomal H^+^-carbohydrate transporter involved in senescence and in the late stages of the autophagy/lysosome pathway. Morpholino knockdown of *spns1* resulted in an accumulation of GFP puncta visualized by confocal microscopy in live embryos and was also observed in *spns1* mutants [10]. Careful characterization using LysoTracker and mCherry-LC3 transgenic fish demonstrated this was due to a block in autophagosome degradation rather than an increase in autophagosome formation. A dual GFP-LC3; mCherry-Lamp1 reporter line recently developed by the same group was used to further elucidate the role of lysosome acidification in senescence [33]. Although analysis was performed *in vivo* in these examples, these studies relied on analysis of single-time-point images to assess autophagosome number and did not exploit the full potential of studying these events in the living organism.

One example of the power of using zebrafish for *in vivo* observations has been in the study of the innate immune response [29]. Transgenic reporters have been used to track individual immune cells throughout the whole organism in response to tissue injury or infection and to study features of swarming and resolution of inflammation [34]. The combination of *in vivo* light microscopy and *ex vivo* electron microscopy imaging opens new directions for studying the role of autophagy in infectious diseases.

Transgenic GFP-LC3 zebrafish infected with *Shigella* have been used to study the process of bacterial clearance *in vivo.* Engulfed bacteria were observed to be sequestered in GFP-positive autophagosomes [35], a finding confirmed by post-mortem transmission electron microscopy analysis. Similarly, during *Mycobacterium marinum* infection in zebrafish, the bacteria were frequently found associated with GFP-LC3-positive vesicles, and these associations were particularly abundant in leucocytes. By correlative light and electron microscopy, the precise location of intracellular bacteria could be elucidated (either free, in autophagosomes or associated with lysosomes) by determining the ultrastructure of GFP-LC3-positive structures [36].

These studies highlight the importance of verifying the properties of the LC3-labelled structures. Although LC3 is the best-established marker to identify autophagosomes, it can also be associated with single membranes on phagosomes within macrophages and other phagocytic cell types where it functions in a process called LC3-associated phagocytosis (LAP) [37]. In this instance, following receptor-mediated phagocytosis, LC3 is recruited to the single-membrane phagosomes using the same conjugation machinery as is involved with macroautophagy. Therefore, within immune cells, careful interpretation of LC3 puncta is required as it may not only detect autophagosomes, but also LC3-labelled phagosomes and correlative light and electron microscopy may be critical in differentiating these processes.

Although these transgenic reporters are powerful tools for studying autophagic processes *in vivo* or in primary cultures, there are important caveats to consider. GFP-LC3 was initially described to localize exclusively on autophagic membranes. However, GFP-LC3 protein can aggregate in an autophagy-independent manner without being conjugated to PE leading to misinterpretation of the results, especially during transient expression of the transgene [26]. For example, GFP-LC3 can be seen to localize with intracellular protein aggregates like huntingtin inclusions in autophagy-null cell lines, suggesting that GFP-LC3 puncta do not always represent autophagic structures and therefore LC3 fluorescent localization should be carefully interpreted. Tanida and colleagues proposed the use of mutant fluorescent LC3 (the human mutation LC3DG), which cannot be lipidated as negative control [38], and as described below, transgenic reporters using this control have now been developed [39].

Since fluorescently tagged-LC3 labels the surface of all autophagic structures, from the formation of the phagophore to the autolysosome, no conclusions can be made about autophagy flux or dynamics by simply measuring the number of puncta. An increase in GFP-LC3 puncta may occur as a result of an increase in autophagosome formation but also could be the consequence of an impairment of autolysosome formation [5]. In cell culture, the inhibition of vacuolar acidification and consequent inhibition of lysosomal activity by bafilomycin A1 (Baf) treatment is commonly employed as a tool to investigate changes in autophagic flux [40]. Such treatment prevents the downstream clearance of autophagosomes and allows a comparison of number of puncta in the presence or absence of lysosomal degradation [5]. *In vivo*, chloroquine or ammonium chloride treatments may be employed to reduce vacuolar acidification, although these treatments are likely to be toxic at saturating concentrations and therefore, at best, can only be considered to be a partial lysosomal block. Such an approach has been used to measure cardiac autophagic flux *in vivo* in mCherry-LC3 transgenic mice [41]. A clearer differentiation between GFP-LC3 associated with autophagosomes or with acidic lysosomes can be achieved by labelling acidic structures with LysoTracker [7,10] or with the use of additional transgenic lysosome markers such as mCherry-Lamp1 [33]. If the co-localization of acidic structures with fluorescent LC3 puncta increases with respect to the total number of labelled structures, this may be indicative of an induction in autophagy. However, this may also occur if there is defective lysosomal function causing delayed LC3 degradation, for example, as observed when components of the chaperonin complex are depleted [42].

A further important consideration is the degradation of GFP-LC3, which can generate free GFP fragments that may accumulate depending on the acidity of the lysosomes and degradative capacity of lysosomal compartments [43]. In cell culture, LC3 was found to be degraded faster than GFP from GFP-LC3 since GFP degradation requires high lysosomal acidification. Starvation, rapamycin or incomplete suppression of autophagy by low doses of inhibitors of lysosomal acidification such as chloroquine (CQ) or Baf also led to higher levels of free GFP fragments from GFP-LC3 in several mammalian cell lines expressing GFP-LC3 [44] and similarly in the liver of GFP-LC3 transgenic mice following CQ treatment [43]. However, it is important to note that this phenomenon and its utility varies in different mammalian cell types and cell lines, and this method has not been widely used in mammalian systems.

## 3. Dual fluorophore probes

**Figure 3.**
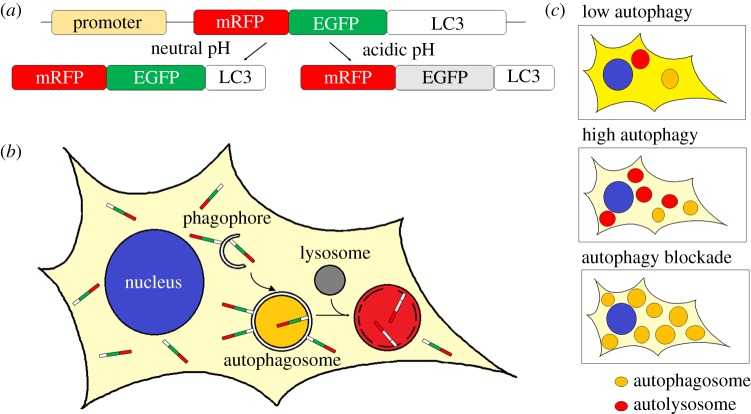
Schematic diagram of the tandem mRFP-EGFP-LC3 reporter to monitor autophagic flux. (*a*) Representation of the reporter construct mRFP-EGFP-LC3 and the behaviour of the encoded protein under different pH conditions. Under neutral pH conditions, both EGFP and RFP fluorescence is observed. Under acidic pH conditions, EGFP fluorescence is quenched and only red fluorescence is observed. (*b*) mRFP-EGFP-LC3 labelling during autophagosome biogenesis, maturation and degradation. Unlipidated mRFP-EGFP-LC3 remains in the cytoplasm (light yellow) whereas lipidated mRFP-EGFP-LC3 is recruited to both inner and outer membranes of phagophores and double-membrane autophagosomes. During these steps of autophagosome formation, the fluorescent signal of both fluorophores, mRFP and EGFP, is visible and vesicles appear as yellow puncta. Autophagosomes eventually fuse with lysosomes to form autolysosomes. Under these acidic conditions, the contents within the inner membrane are eventually degraded. The green fluorescent signal from EGFP is quenched in the acidic lysosomal conditions whereas the mRFP signal remains, resulting in red autolysosomes. (*c*) Representative images of a cell expressing mRFP-EGFP-LC3 with different levels of autophagy. The combination of green and red fluorescent signals from unlipidated mRFP-EGFP-LC3 results in a yellow background in the cytoplasm of the cells. The intensity of this yellow may change dependent upon changes in the autophagy flux. Under low autophagy conditions, most of mRFP-EGFP-LC3 remains unlipidated resulting in a yellow background and only a few yellow or red vesicles (autophagosomes and autolysosomes) are seen. After autophagy induction, many new autophagosomes form and are labelled with lipidated LC3. These rapidly fuse with lysosomes. This can be observed as an increase in the number of total vesicles and the ratio of red:yellow vesicles as well as reduced yellow background. When autophagic flux is blocked, autophagosome formation may still occur. In this scenario, autophagosomes and autolysosomes accumulate but cannot be degraded and can be observed as yellow puncta. The continuous lipidation of mRFP-EGFP-LC3 as new autophagosomes form reduces the yellow background of the cytoplasm.

Since the GFP fluorescent signal is quenched in the acidic environment of autolysosomes, this limits the utility of this reporter for tracking vesicles during the autophagic process. To overcome these limitations, a tandem fluorescent tagged-LC3 was developed and initially characterized *in vitro* [45]. The fluorescent proteins GFP and mRFP have different properties under acidic conditions. Kimura and collaborators [45] showed that using a tandem-tagged mRFP-GFP-LC3, GFP fluorescence (pKa 5.9) is quenched in the acidic environment of the lysosomes, whereas the red fluorescence from the mRFP tag (pKa 4.5) is maintained due to its different sensitivity to pH. As a consequence, GFP channels and mRFP channels of the same labelled cells showed different distribution patterns of puncta. The development of the tandem fluorescent mRFP-GFP-LC3 has been widely used *in vitro* to study the mechanisms regulating the maturation of autophagosomes and the fusion to lysosomes in the degradative process. Owing to this pH-dependent quenching of the GFP-LC3 fluorescence, only mRFP-LC3 can be detected in autolysosomes (i.e. these appear red only), whereas autophagosomes can be visualized by both fluorophores (i.e. these appear yellow) (figure 3).

The first *in vivo* mouse model expressing mRFP-GFP-LC3 was generated by Li and colleagues in 2014 [46]. Expression of the LC3 tandem reporter was ubiquitous, which allowed a better understanding of the dynamics of autophagy *in vivo* under stress conditions, such as starvation and disease. In these RFP-EGFP-LC3 mice, autophagic vacuoles were visualized as RFP- and EGFP-positive puncta, similar to *in vitro* observations in cells expressing the same construct. The model was first used to evaluate the role of autophagy in ischemia-reperfusion injury in the kidney using primary cell culture. In addition, primary cortical neurons from an independently generated mouse line have been used to investigate the interplay between chaperone proteins and autophagy [42]. Tandem construct mCherry- or RFP-GFP-LC3 have also been used in zebrafish. Transient expression of RFP-GFP-LC3 in zebrafish was used to investigate the autophagy pathway in the clearance of mycobacterium infection. Treatment with carbamazepine was shown to improve the clearance of mycobacterial infection *in vivo* and increase autophagic flux in larvae zebrafish [47]. Stable transgenic zebrafish expressing mCherry-GFP-map1lc3b have also been used to evaluate the autophagic and late endosomal trafficking pathways in the cone photoreceptors of *synJ1*-deficient zebrafish [48–50]. Live time-lapse confocal microscopy revealed an increase in the formation of autophagosome precursors and a defect in autophagosome maturation *in vivo* in *synJ1*-deficient zebrafish, resulting in the accumulation of autophagosomes. Modulation of the PI(4,5)P2 regulator, Arf6, by expressing a constitutively active mutant of Arf6, rescued the defects seen in cones of synJ1-deficient fish. These results suggest that Arf6a modulates positively the levels of PI(4,5)P2, a substrate for SynJ1, and hence that both Arf6 and SynJ1 play a role in the same pathway to regulate autophagy in cone photoreceptors [49]. These studies highlight the potential of the zebrafish model to characterize aspects of vesicle transport *in vivo*.

However, as with the analysis of GFP-tagged LC3, there are additional factors to be considered when using this tandem red-green fluorescent LC3 fusion protein. First, the red and green fluorescence from unconjugated LC3 exists in the cytosol of all cells. When autophagic flux is low, this background is higher. As the LC3 becomes conjugated and more puncta appear, the background fluorescence decreases (figure 3). Identifying puncta against this fluorescent background is challenging and care must be taken in quantifying the number of autophagosomes in conditions where the background fluorescence changes. Second, due to the pH-sensitivity of the GFP signal, reduction in the green signal may depend not only on the enzymatic degradation of GFP itself but also the speed at which the lysosomal content acidifies [6]. Thus, what one is formally assessing is the number of unacidified versus acidified LC3-containing vesicles, which may not always be the same as the number of autophagosomes (prior to lysosome fusion) versus autolysosomes.

**Figure 4.**
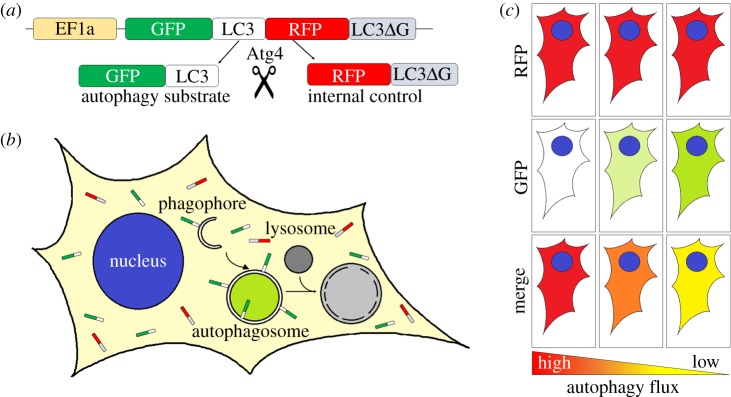
Schematic diagram of the GFP-LC3-RFP-LC3DG reporter to measure autophagic flux. (*a*) Schematic diagram of the GFP-LC3-RFP-LC3DG reporter construct. The GFP-LC3-RFP-LC3DG protein is cleaved by ATG4 resulting in the release of GFP-LC3 and RFP-LC3DG in equimolar amounts. (*b*) GFP-LC3 becomes lipidated and binds to autophagosomes and autophagosome precursors, and can be visualized as green vesicles (puncta), whereas unlipidated RFP-LC3DG remains in the cytoplasm. The GFP signal is quenched when autophagosomes fuse to lysosomes to form autolysosomes. The green signal can therefore be used as a marker for phagophores and autophagosomes, but autolysosomes are not labelled. (*c*) Representative images of a cell expressing GFP-LC3-RFP-LC3DG with different levels of autophagy. The unlipidated RFP-LC3DG is released as an internal control at the same rate and amount as GFP-LC3 and always remains cytosolic. Levels of red signal are independent of autophagy degradation and remain unchanged upon autophagy perturbation. GFP-LC3, however, can be found unlipidated free in the cytoplasm or lipidated, hence bound to autophagic membranes, and therefore susceptible to autophagy degradation. Under high levels of autophagic flux, GFP-LC3 becomes lipidated and degraded, and thereby the level of green signal is reduced. When autophagy is blocked, the accumulation of unlipidated GFP-LC3 and the lack of degradation of the lipidated form results in an increase in the GFP signal. The ratio of the GFP:RFP (i.e. the green signal from GFP-LC3 and the unchanged mRFP-LC3DG) is then used to measure the rate of autophagic flux.

The development of a new generation of fluorescent probes may help with some of these difficulties. A new dual-fluorescence probe was recently generated by the Mizushima group comprising GFP-LC3-RFP-LC3DG [39]. The expression of the construct results in a protein that is cleaved by Atg4 proteases resulting in the equimolar amounts of two separate fluorescently tagged proteins; GFP-LC3 and RFP-LC3DG. RFP-LC3DG is a mutated form of LC3, which cannot be conjugated (figure 4). It is therefore unable to attach to autophagic membranes, remaining in the cytosol and hence can be used as an internal control. However, GFP-LC3 can be lipidated and attaches to the autophagosome membrane. GFP-LC3 on the inner autophagosome membrane is degraded by autophagy whereas on the outer membrane it is deconjugated by Atg4 and returns to the cytosol. The ratio of GFP/RFP can, therefore, be used as a measurement of autophagic flux as it assesses LC3 degradation via a conjugation-dependent route (i.e. autophagy). However, as only a small proportion of the protein is degraded, the windows of detection are limited.

Mice and zebrafish expressing GFP-LC3-RFP-LC3DG were developed to evaluate autophagic flux in different tissues and validated to confirm that the reporter responds appropriately to drug-induced autophagy upregulation [39]. Although the transgene was detected in several tissues by western blotting in mice, only skeletal muscle showed sufficient levels of expression for fluorescence analysis. Post-mortem analysis of muscle sections was used to evaluate fed versus fasted conditions. Interestingly, their findings suggest that slow and fast twitch muscle fibres have different levels of basal autophagy [39].

The use of other fluorescent tandem reporters with different pH-sensitivities, such as mWasabi (pKa at 6.5 versus pKa 5.9 for GFP) leads to a faster loss of fluorescence in the autolysosomes [51] and may be a better tool for tracking autophagy flux *in vivo*. Both mTagRFP and mWasabi-LC3 are much brighter than mRFP/mCherry and EGFP fluorescence. mWasabi is also more acid-sensitive than EGFP and hence more easily quenched in the acidic environment of autolysosomes [52]. In addition, the pKa of mTagRFP (4.0) is lower than that of mRFP (4.5), suggesting that mTagRFP is more stable than mRFP in acidic conditions [53]. These characteristics make discrimination of autolysosomes and autophagosomes more accurate than other fluorophores and were used to investigate the dose-dependent effect of autophagy inducers in the autophagic flux in cells [51]. However, no *in vivo* models have been created using this construct. Similarly Rosella, a tandem reporter of the fast maturing red fluorescent protein dsRed.T3 with GFP, has been successfully used to track labelled cytosolic proteins, mitochondria or the nucleus to the autophagic vacuole in yeast [54,55]. Rosella-LC3 and Mito-Rosella biosensors have been developed and characterized in HeLa cells [56]. These authors reported that transgenic mouse models for Rosella-LC3 and Mito-Rosella biosensors were being developed to measure mitophagy and autophagic flux in different tissues *in vivo*, although no further data have been published to date.

## 4. pH-sensitive probes

In contrast to the use of dual fluorophores to label LC3, new approaches have been developed in recent years which allow one to measure autophagic flux using a single fluorophore. dKeima, a coral-derived fluorophore, has a bimodal excitation spectrum (438 and 550 nm) with an emission spectrum peak at 620 nm [57]. The different excitation wavelengths correspond to the neutral and ionized states of the chromophore with the neutral state (438 nm excitation) predominant at neutral/high pH and the ionized state (550 nm excitation) more abundant at low pH. Therefore, dual-excitation ratiometric imaging (438/550 nm) can be used to determine the environmental pH [58]. In cell culture experiments, dKeima was demonstrated to be delivered to lysosomes via the autophagic pathway and was observed to accumulate inside the lysosomal compartments because it is relatively resistant to degradation by lysosomal proteases [58]. Hence ratiometric imaging over time can be used to monitor the maturation of autolysosomes and therefore autophagic flux. Furthermore, since the emission spectrum of dKeima peaks at 620 nm, this probe can be simultaneously imaged with green fluorophores (e.g. EGFP-LC3) without cross-detection or excitation [58].

In addition, Keima can be targeted to either proteins or organelles. For example, Keima targeted to mitochondria (Mito-Keima) has been used to evaluate mitochondrial autophagy (mitophagy) in cell culture [58]. Mito-Keima has also been used in mice via intravenous injection of adeno-associated virus (AVV9) harbouring either Mito-Keima or Lamp1-YFP (yellow fluorescent protein) to evaluate mitophagy in cardiomyocytes of the adult heart [59]. Confocal imaging of thin slices of the heart showed Lamp1-YFP dots colocalizing with acidic Mito-Keima (561 nm) after 48 h starvation of the animals, suggesting that the lysosomal degradation of mitochondria is stimulated after fasting.

## 5. Labelling autophagic substrates

**Figure 5.**
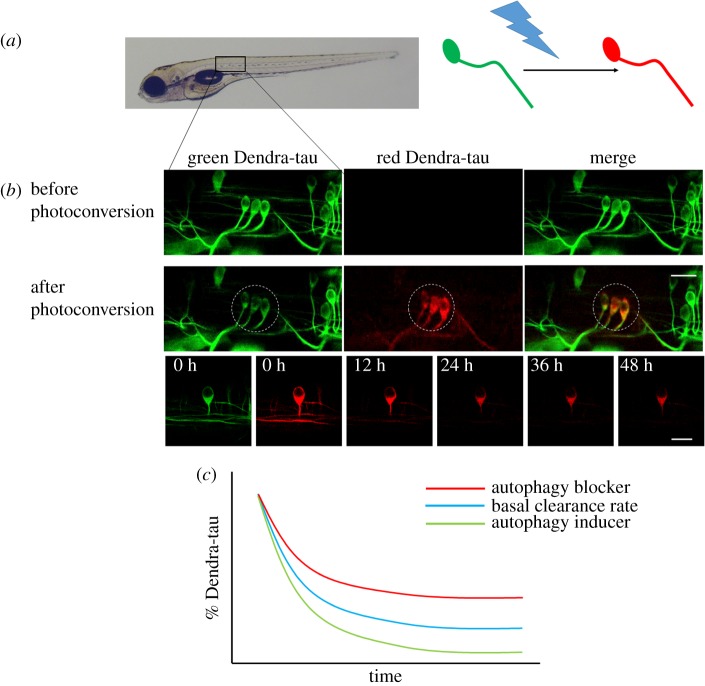
Measuring autophagy substrate clearance *in vivo*. (*a*) Zebrafish were generated which express the fluorescent, photoconvertible protein Dendra tagged to human tau, a known autophagy substrate. The green fluorescent Dendra protein can be photoconverted to a red fluorescent protein by exposure to 405 nm light. (*b*) Mosaic expression of the transgene allows individual neurons in the spinal cord to be identified and selected for photoconversion. Images of the same neurons were taken before and immediately after photoconversion and then at 12 h intervals. The amount of red fluorescent signal was quantified at each time point and used to calculate the clearance of tau protein. (*c*) Schematic diagram of the clearance kinetics of tau in response to manipulation of autophagic flux. Treatment with autophagy inducers (green) accelerates the clearance of tau protein whereas treatment with autophagy blockers (red) slows the clearance kinetics.

An alternative approach is not to measure autophagosomes *per se* but to measure the clearance of autophagic substrates. Tau is a microtubule-associated protein which is known to be an autophagy substrate [60]. Zebrafish models have been developed expressing a transgenic construct comprising human tau tagged with the photoconvertible fluorescent protein Dendra. The fluorescently tagged tau protein is visible as green fluorescence but this can be converted to a red fluorescent protein by exposure to 405 nm wavelength light. This conversion labels a steady-state pool of tau protein allowing clearance kinetics to be measured without being confounded by new protein synthesis (since newly formed protein will be green). This method has been used to assess both genetic modifiers of tau clearance [8] and also to assess clearance of wild-type and mutant forms of tau in response to autophagy stimulus [9]. Such studies have provided the first observations of substrate clearance in neurons *in vivo* (figure 5). This approach has also been used to study the clearance of mutant huntingtin in cell culture [61]. Although clearance of substrates is probably affected by both the proteasome and autophagy, the use of proteasome blocking agents (e.g. MG132) and lysosomal acidification inhibitors (e.g. Baf, CQ or ammonium chloride) allows discrimination between the two clearance pathways and an assessment of the relative contribution of each.

## 6. Future directions/conclusion

To date, much of our understanding of autophagosome formation, trafficking and degradation have come from work in cell lines or in primary cell culture. The elegant work of the Holzbaur group in studying trafficking in primary neurons has revealed important aspects of autophagosome trafficking and biogenesis [62–64]. Given the tools described here, and the advances imaging techniques, it is likely that we now have the ability to investigate many of these processes *in vivo*. Indeed, such approaches have been applied to the *in vivo* trafficking of mitochondria [65–67]. Caveats remain about the fidelity of transgenicly labelled proteins, since these protein-tags are expressed in addition to the endogenous protein, typically at higher levels than the endogenous protein, and are not controlled by the endogenous promoter. However, chromobody labelling may be one approach that can be used to overcome this. These are small antigen recognizing elements (nanobodies) fused to fluorescent reporters and have been used to label actin cytoskeleton and cell-cycle-associated proteins in zebrafish [68]. In addition, recent advances in CRISPR- and TALEN-mediated knock-in methodologies [69,70] suggest that in future it may be possible to specifically add tags to endogenous proteins. Therefore, although we have not yet exploited the full power of the transgenic, genomic editing and imaging technologies, the tools are now available to allow us to better investigate the process of autophagy in health and disease within living tissues. Since autophagy impacts on a diverse range of pathological conditions, such as neurodegeneration, infection and cancer, the ability to visualize how autophagic flux is affected *in vivo* in such disease states will provide valuable information on which steps of the pathway can be manipulated for therapeutic benefit.

## Data accessibility

This article has no additional data.
